# Integrins and Exosomes, a Dangerous Liaison in Cancer Progression

**DOI:** 10.3390/cancers9080095

**Published:** 2017-07-26

**Authors:** Mayra Paolillo, Sergio Schinelli

**Affiliations:** Department of Drug Sciences, University of Pavia, Viale Taramelli 14, Pavia 27100, Italy; sergio.schinelli@unipv.it

**Keywords:** cancer stem cell, tumor niche, exosomes

## Abstract

Integrin activity and function is classically related to the bi-directional regulation of cell-extracellular matrix (ECM) contacts that regulate a number of cell pathways linked to cell adhesion, cell detachment from ECM, cell migration, and anoikis. Interestingly, emerging data continue to uncover new roles for integrins in cancer-relevant pathways, particularly concerning the regulation of immune cell activity in the tumor niche, like myeloid cell differentiation and function and, very recently, the regulation of metastatic processes by exosomes. Exosomes are deeply involved in cell-cell communication processes and several studies have shown that integrins found in tumor-associated exosomes can promote cancer progression by two novel cooperative mechanisms: horizontal transfer of integrin transcripts as vescicle cargo, and selection of target tissues to form new tumor niches during metastatic spread by integrins carried on the exosome’s surface. In this review we will discuss mounting evidence that contribute to the development of a new picture for integrins in cancer, highlighting the role of integrins in the processes that leads to tumor niche formation. In particular, the role of the periostin pathway in the recruitment of tumor-associated macrophages, and the proposed contribution of exosome-derived integrins in the metastatic spread will be discussed. Finally, in light of the above considerations, an evaluation of integrins as possible therapeutic targets will be conducted.

## 1. Integrins in Cancer—an Overview

The physiological role of integrin receptors in the life of a cell is complex, involving cellular pathways that are interconnected and contribute to cell migration and cell survival; as a consequence, the role of integrins in numerous pathologies appears to be very relevant. The role of integrins in cancer has been closely monitored and investigated ever since the first reports documented an overexpression of certain integrin receptors in tumor samples, compared to non-tumor surrounding tissues. The most interesting aspect, in fact, is that some integrins are differentially expressed in normal and cancer cells: they have been found to be expressed at very low levels in most adult epithelial cells, while they are overexpressed in some solid tumors [1].

Integrins belong to a large family of cell adhesion receptors and consist of two transmembrane glycoproteins, α and β that, outside the cell, interact with the extracellular matrix (ECM) proteins and are primarily involved in cell adhesion mechanisms. Cell adhesion is a necessary condition for healthy cell life and dysfunction in this process leads to most common human diseases, including immuno-mediated diseases, coagulation disorders, and cancer progression. ECM ligands binding to integrins leads to integrin clustering and the subsequent activation of a downstream non-receptor tyrosine kinase, focal adhesion kinase (FAK), which initiates a series of signaling pathways. Consolidated evidence has demonstrated that FAK regulates cell migration via modulation of focal adhesion turnover, and modulates actin cytoskeleton polymerization and lamellipodia protrusion [2]. Moreover, FAK signaling, via the IP3 pathway, leads to Akt activation and inhibition of pro-apoptotic pathways. Taken together, these studies highlight two features regulated by integrins that turn to be essential to cancer cell survival and cancer progression: regulation of cell adhesion and cell migration mechanisms and regulation of survival signals.

A number of studies have identified several integrins that play a key role in cancer progression, providing the rational basis for the development of integrin antagonists as potential therapeutic tools in cancer [3]. Expression of the integrins αvβ3, αvβ5, α5β1, α6β4, α4β1, and αvβ6 is correlated with disease progression in various tumor types. Studies on non-small cell lung cancer (NSCLC) have emphasized the role of integrins in the development and progression of this tumor type, thus prompting the use of Cilengitide, the first integrin antagonist to be synthesized, in clinical trials in association with chemotherapeutic drugs. In addition, the finding that αvβ3, α5β1, and αvβ6 integrins have been found in lung cancer lymph node metastasis, together with the observation that bone metastatic cells in advanced prostate cancer express αvβ3 integrin, led to the proposal that specific integrin expression could also represent a reliable prognostic marker of overall survival in some cancer types [4,5,6].

## 2. Integrins and Metastasis: State of the Art

Several cancer cells undergo complex processes that lead to metastasis formation: these cells detach from the primary tumor mass, infiltrate surrounding tissues, and reach blood or lymphatic vessels. These migrating cells, traveling through the bloodstream, eventually colonize in a vascular place by adhering to endothelial cells, or cross the vascular barrier to begin the process that leads to the pre-metastatic niche formation in the target organ parenchyma. Thereafter, cancer cells grow and invade the tissues in different ways, such as expansive growth, multicellular migration, and individual cell migration [7].

On the other side, the host tissue re-organizes its structure and vasculature, recruiting stromal cells, such as fibroblasts, endothelial cells, and tumor-associated macrophages, which sustain the tumor growth by producing and releasing extracellular matrix proteins, cytokines, and growth factors [7]. The ECM composition can also be modulated by exosomes released by cancer cells to create the right conditions for the adhesion of migrating tumor cells. In particular, exosomes isolated from a rat pancreatic carcinoma cell line model have been found to modify the ECM of other rat tissues, facilitating tumor cell attachment [8].

Cancer stem cells, CSC, a subpopulation of cancer cells that retain stemness features and are resistant to anoikis, have been identified as being responsible for migration processes that lead to the formation and colonization of the metastatic tumor niche [9]. Most adult tissues, indeed, maintain some aspect of this migratory capacity through the ability to generate an epithelial to mesenchymal transition (EMT)-like process during wound healing, tissue regeneration, and organ fibrosis. During the EMT process, identified as one of the earliest steps of solid tumor progression, epithelial cells undergo a developmental switch leading to decreased adhesion and loss of cell polarity, increased proliferation, motility, and invasiveness. The consequence is that epithelial cells acquire a stem-like phenotype. These changes are associated with the downregulation of epithelial cell surface markers and cytoskeleton components (E-cadherin, zonula occludens [ZO]-1, claudins, occludins, cytokeratins) and the upregulation of mesenchymal markers (vimentin and α-smooth muscle actin) together with extracellular matrix components (collagens and fibronectin) [10].

CSC are supposed to activate their migration program through the process of EMT. Indeed, a variety of mechanisms has been demonstrated to contribute to CSC functions; several microRNAs (miRNAs) may participate in the activation of CSC-like activities and loss of miR-124, which regulates proliferation, for instance, has been shown to promote glioma formation [11].

KLF4, a transcription factor and oncogene which is able to switch somatic cells into stem-like states, has potent oncogenic activities in mammary tumorigenesis and is likely involved in maintaining stem cell-like features in tumor cells [12].

Expression of phosphatase and tensin homolog (PTEN) is also thought to be critical for the establishment of a stem-like state [13], and its loss correlates with prostate cancer metastasis and progression. Recent studies linking the RAS/MAPK pathway with PTEN deletion demonstrated the development of an EMT state in the prostates of experimental animals [14]. Therefore, assuming that CSC play a leading role in metastasis spread, integrins appear to be the missing link between EMT and CSC.

Among the wide variety of cellular pathways which contribute to EMT, in fact, integrins and transforming growth factor (TGF-β) efficiently cooperate to drive tumor cells towards EMT [3,15]. The TGF-β activation pathway, in fact, requires integrin binding to the TGF-β precursor peptide, which includes in its structure Latency Associated Peptide (LAP) and Latent TGF-β-Binding Protein (LTBP) peptide sequences. The LAP peptide, in turn, contains in its sequence an Arg-Gly-Asp (RGD) motif that is recognized by RGD-binding integrins αvβ3, αvβ5, α5β1, and αvβ6 expressed on the cell membrane; the resulting binding generates a stretch force that leads to breaking of the precursor peptide, thereby releasing the active form of TGF-β. In this interaction, RGD-binding integrins actually represent the missing link between EMT induction by TGF-β and metastatic process onset.

The EMT phenotype has been detected in different solid tumors with high metastatic potential, like lung, breast cancer, and melanoma [16,17]; furthermore, in lung cancer, the expression of EMT markers is associated with prognosis. In light of this evidence, RGD-based integrin antagonists were suggested to be potentially useful tools to inhibit metastatic diffusion [18,19].

## 3. Exosomes and Cancer

Exosomes are extracellular vesicles with a diameter of 30–130 nm that are secreted from cells and are thought to provide a means of extracellular communication by vehiculating macromolecules, such as proteins, miRNA, RNA, and DNA, from cells to other target tissues [20]. The consequences of this biological transfer can be very different, according to the cell type of origin, the target cell, and the molecules contained in exosomes. Initially, exosomes and extracellular vesicles were found to transfer oncoproteins and nucleic acids from cells infected by virus to other surrounding cells, thus creating the favorable conditions for tumor progression induced by viruses [21]. Several studies have further confirmed that systemic circulation of exosomes can play a role in the formation of the tumor niche [22]. However, when exosomes reach target cells, before releasing their content, they must fuse with the target cell membrane. The mechanisms that regulate such fusion are not entirely known because fusion with the cytoplasmic membrane appears to be differently regulated depending on the target cells [23].

Exosomes’ molecular features are thought to reflect the trait of the cells of origin and the finding that exosomes circulate in body fluids, such as blood and urine, prompted the idea that they could be valuable diagnostic markers for various diseases, including cancer. This hypothesis was further supported by evidence showing increased levels of circulating exosomes in cancer patients and their potential use as prognostic markers appears to be a possibility [24].

It has been reported, in fact, that miRNAs found in exosomes isolated from the serum or urine of ovarian carcinoma patients at various stages were not detectable in the normal controls, representing promising diagnostic tools [24]. In another very recent study a set of miRNAs associated to exosomes isolated from colon carcinoma patients was identified as a possible valuable tool to identify patients at higher risk of liver metastasis relapse. In addition, in this study the authors found that exosome-associated miR-328 may play an important role in modulating the microenvironment for the anchoring of metastatic cells to liver tissue [25]. Similarly, in osteosarcoma patients’ serum, an exosome-associated miRNA was identified that correlated well with patient prognosis, having favorable sensitivity and specificity compared with serum alkaline phosphatase [26].

Taken together, these findings indicate that miRNAs of circulating tumor-derived exosomes could potentially be used as diagnostic markers or be extended to the screening of asymptomatic populations.

However, this horizontal transfer operated by exosomes has profound effects on target cells and implies the ability of exosomal content to deeply modify, or even re-program, the target cell genome: exosomes secreted by breast cancer cells vehiculating several pre-miRNA, in fact, have been found to induce genotype and phenotype changes of target non-malignant cells [27]. More recently, efforts have been directed at characterizing the surface components of exosome membranes; exosomal proteins, in fact, maintain their functional activity and can interact with target cells.

Exosomes isolated from Chronic Lymphocytic Leukemia (CLL) patients have been shown to switch endothelial and mesenchymal stroma cells into cancer-associated fibroblasts to sustain leukemic cell survival in vitro. Additinoally, circulating extracellular vesicles from CLL patients were shown to stimulate bone marrow stromal cells, inducing the production of B-cell survival factor hypoxia-inducible factor-1a [28]. Interestingly, the composition of surface proteins expressed by exosomes and other larger vesicles differs from the expression of the same proteins found in the releasing cells grown in vitro, suggesting a sort of commitment of exosomes towards the modification of other tissue environments.

Among the proteins whose expression has been found to vary between origin cancer cells and the released exosomes in different cell models, tetraspanins have recently attracted interest. Tetraspanins are surface proteins that cross the membrane four times and whose biological role has not been fully clarified yet. They are known to assemble with a variety of receptors, thus modulating their ability to cluster and their activation; the best known tetraspanin partners are integrins, particularly α3β1, α4β1, and α6β1 [29]. For this reason, a cooperative role for integrins and tetraspanins in targeting exosomes to selected tissues has been suggested; tetraspanins, in fact, have been found in exosomes derived from different cancer cell types, and exosomes released from a rat pancreatic cancer cell line transfected with tetraspanin-8 (Tspan8) and β4 integrin were found to preferentially bind to spleen cells, and lung and kidney tissues [30]. This selectivity was suggested to depend on tetraspanin-β4 ability to bind target cells [30], however, the relationship between tetraspanins and exosome-associated integrin activity, though intriguing, is still unclear.

A recent paper shed further light on this issue: an adhesion protein found in tumor exosomes released by pancreatic and colorectal cancer-initiating cells, CD44v6, appears to contribute to tumor progression by cooperating with α6 and β4 integrins, leading to regulation of Tspan8 expression and the enhancement of cell migration and invasion in recipient cells [31].

Other evidence has shown that endothelial cell-derived exosomes regulate the phenotype of hepatic stellate cells (HSCs); these exosomes have been found to contain fibronectin, a ligand for cell surface integrins, specifically RGD-binding integrins, and that this interaction can mediate exosome adhesion to HSCs. Interestingly, binding of target cell integrins to exosomal fibronectin binding activates integrin recycling and exosomal internalization [32].

## 4. Exosomes and Integrins, the Dangerous Liaison

The role of integrins in cancer growth and metastasis spread has been highlighted and a variety of cellular mechanisms involving integrin receptor activation has been observed in cancer cells, in the tumor niche and in the vascular compartment. Evidence is accumulating concerning the role of integrins, especially αvβ3, in the induction of the mesenchymal state in CSC and in their adhesion and survival in the tumor niche [33]. Additinoally, integrin β4 has been indicated as a marker of mesenchymal carcinoma cell populations that can be used as a prognostic biomarker to identify the more aggressive subtypes of mesenchymal carcinoma cells in triple-negative breast cancer [34].

In this quite complicated picture, integrins appear to be involved in almost every step of cancer progression and the finding that integrins are expressed in CSC shed further light on the metastatic seeding process.

Nevertheless, researchers still have to face significant, unanswered questions: how does CSC recognize the target site for metastases and, even more importantly, which mechanisms initiate the metastatic niche formation? The idea that some soluble factors, probably released by primary tumor cells, might be the drivers of this process was somehow suggested in several studies, especially considering the emerging role of exosomes released by cancer cells [35]. However, some studies published in 2015 and 2016 contributed considerably to clarifying this issue and the links between exosomes and integrins were observed, as summarised in Table 1.

In a recent study [36] exosomes from prostate carcinoma cells, PC-3, were isolated and subjected to quantitative proteomic analysis; the protein expression pattern was found to be different in taxane-sensitive and taxane-resistant (PC-3R) cells. Increased levels of β4 integrin and vinculin (VCL) were found in PC-3R cells and in the exosomes released by these cells, compared with PC-3 cells. Interestingly, the authors found that the knockdown of β4 and VCL genes in PC-3R cells reduced cell migration and invasion, but did not modify the proliferation rate and taxane resistance. The authors, therefore, suggest that the exosomal content of β4 and VCL could represent a predictive marker for taxane resistance in prostate carcinoma patients; interestingly, these data also highlight the possibility that the horizontal transfer of β4 and VCL to other cancer cells could confer or enhance migratory skills of the target cells.

αvβ6 is an RGD-binding protein whose overexpression has been reported to promote metastasis, and to be related to cancer progression and poor clinical prognosis in several tumor types [40,41]. Like other RGD-binding integrins (see above), αvβ6 binds the TGF-β precursor peptide, thereby triggering the EMT process. Interestingly, this integrin was also found to be involved in TGF-β mediated matrix metalloproteinase-2 (MMP2) activation, probably representing the promoter of the osteolytic process in PrCa bone metastases [42].

However, the dangerous liaison between αvβ6 integrin and exosomes was demonstrated in a study showing that exosomes released from PrCa cells were able to transfer αvβ6 to other non-expressing tumor cells. Even more importantly, this study showed, for the first time, that the target cells, after the fusion with αvβ6 containing exosomes, also acquired the cellular functions related to its expression [37]. Particularly, αvβ6 transfer to neighboring tumor cells resulted in enhancement of cell adhesion and cell migration by a paracrine mechanism.

In another study, αvβ3 integrin was found in exosomes released by metastatic prostate cancer cells and in tumor-bearing mice blood [38]. The αvβ3 carrying exosomes were efficiently internalized by non-tumor cells and, as a functional correlate, an increase of αvβ3 expression in target cells, together with the increase of cell adhesion and cell migration, was observed. This study strongly supports the hypothesis that exosomal integrin transfer results in the concomitant gain of function by the target cell.

Another study, published a few months before this, addressed the issue of organ-targeted metastases; the authors investigated whether molecules present on tumor-derived exosomes could drive them to specific organs [39]. The exosomes were isolated from cell lines of different cancer types (osteosarcoma, rhabdomyosarcoma, Wilms tumor, melanoma, breast, colorectal, pancreatic, and gastric cancers) and selected on the basis of their trend to metastasize to specific organs like brain, lung, or liver. The proteomic profile of exosomes released by several cancer types was analyzed and exosomes were successively injected in nude mice to follow their localization.

Tumor-derived exosomes carried specific integrins which directed adhesion to distinct target tissues. Particularly, exosomal integrins were found to initiate organ colonization by fusing with target cells of specific tissues and leading to metastatic niche formation. The authors suggest that exosomal integrins interact with cell-associated extracellular matrix in specific organs, thus identifying the target organ for metastatic spread.

In detail, the striking finding is that exosomes expressing the αvβ5 integrin were found to specifically bind to Kupffer cells, thus identifying the liver as the target organ for pre-metastatic niche formation. Similarly, exosomal α6β4 and α6β1 integrins were found to bind to lung fibroblasts and epithelial cells, directing metastatic seeding to the lung [39].

A different mechanism was suggested for exosomes that specifically targeted bone: these exosomes, in fact, were found to contain low integrin levels and did not fuse with lung parenchymal cells; on the other hand, they were able to induce vascular leakiness in the lung and the authors hypothesize that induction of vascular leakiness may be the first event mediated by exosomes during the metastatic cascade in bone.

## 5. Conclusions

In conclusion, exosomal integrins appear to play two different roles: by the horizontal transfer of the receptor itself, they can make other cancer cells acquire skills that that they normally do not display, like migratory and infiltrative behavior. Other evidence shows that the different expression of exosomal integrins can direct metastatic niche formation and, eventually, organ-specific metastasis spread.

Taken together these data strongly underline that integrin receptors could represent possible valuable targets to limit metastatic spread, and further studies are warranted to verify if integrin inhibition could be a way toward this target. Integrin antagonists used in clinical trials, together with classical anticancer drugs, did not give significant therapeutic advantages. Two possible explanations could be provided for this failure: first, integrin-targeting drugs may display moderate affinity in vivo for integrin receptors, as reflected by the doses used in clinical trials [43]. Secondly, integrin antagonists might do their best work by inhibiting early metastatic spread and not in advanced cancer stages, when cancer cells are already widespread.

Finally, a caveat should be considered; generally, in the studies dealing with the biological effects of exosomes, micrograms of exosomal proteins are used. This experimental model, though necessary to warrant measurable biological effects is, nevertheless, far from the in vivo exosomal concentration in blood or in other body fluids. Therefore, future studies should be directed to the evaluation of exosomal integrins functions under conditions that more closely resemble the in vivo concentrations.

## Figures and Tables

**Table 1:** Integrins and integrin ligands found in exosomes.

Exosomal Integrins/Exosomal Integrin Ligands	Cell Type/Source	Reference
β4	Pancreatic cancer cells	[30]
α6, β4	Pancreatic and colorectal cancer initiating cells	[31]
fibronectin	Endothelial cells	[32]
β4, vinculin	Prostate carcinoma cells	[36]
αvβ6	Prostate carcinoma cells	[37]
αvβ3	Prostate carcinoma cells	[38]
αvβ5, α6β4, α6β1	Breast and pancreatic cancer cells	[39]
